# Feasibility of guided internet-based cognitive behavioral therapy for patients with anorexia nervosa

**DOI:** 10.1016/j.invent.2022.100504

**Published:** 2022-02-14

**Authors:** Sayo Hamatani, Kazuki Matsumoto, Jumpei Takahashi, Yuki Shiko, Yoshihito Ozawa, Tomihisa Niitsu, Yoshiyuki Hirano, Eiji Shimizu

**Affiliations:** a Research Center for Child Mental Development, Chiba University, Japan; b Learning and Behavior Science, Linköping University, Sweden; c Research Center for Child Mental Development, University of Fukui, Japan; d Laboratory of Neuropsychology, Kanazawa University, Japan; e Department of Child Psychiatry, Chiba University Hospital, Japan; f Biostatistics Section, Clinical Research Center, Chiba University Hospital, Japan; g Department of Psychiatry, Graduate School of Medicine, Chiba University, Japan; h Department of Cognitive Behavioral Physiology, Graduate School of Medicine, Chiba University, Japan

**Keywords:** Anorexia nervosa, Cognitive behavioral therapy, Internet-based intervention, Clinical trial, Feasibility study

## Abstract

**Objective:**

The objective of the present study was to investigate the feasibility of guided internet cognitive behavioral therapy (ICBT) for anorexia nervosa.

**Methods:**

We conducted a prospective single-arm study between January 2020 and March 2021. The intervention was built using videos, web programs, and chat tools. The intervention program was largely based on metacognitive training. Participants performed the self-help program once a week for 12 consecutive weeks. The primary outcome was the global Eating Disorder Examination Questionnaire (EDE-Q) score. Secondary outcomes included clinical symptoms of eating disorders, metacognitive function, body mass index, depression, and generalized anxiety. The main statistical analysis examined whether the EDE-Q score and other outcomes at the end of the intervention differed from the baseline.

**Results:**

Fourteen participants underwent the trial treatment, and 13 completed the intervention. There was a significant reduction in the global EDE-Q score from 3.48 (*SD* = 1.4) to 2.54 (*SD* = 1.5, *p* = 0.02, Cohen's *d* = 0.75) from baseline to post-intervention. Some EDE-Q subscales and body checking questionnaire scale demonstrated statistically significant improvements, with moderate to large effect sizes. Although there was no significant improvement in body mass index, metacognitive function, or depressive symptoms, there was a significant improvement in the severity of generalized anxiety (*M* = −4.0, *p* = 0.01, Cohen's *d* = 0.95). No adverse events were observed.

**Discussion:**

Our findings suggest that guided ICBT for anorexia nervosa is well accepted by female patients and practical as a telemedicine approach that improves symptoms. In the future, tightly controlled randomized controlled trials should be conducted for efficacy verification.

## Highlights

• The Internet-based CBT was feasible for patients with anorexia nervosa.
• No adverse effects were reported by the patients with anorexia nervosa.
• The ICBT improved the severity of eating disorders and anxiety in patients with anorexia nervosa.
• A randomized controlled trial should be needed to examine the effectiveness of ICBT for anorexia nervosa in the future.

## 1 Introduction

Anorexia nervosa (AN) is a severe psychiatric disorder that primarily affects adolescents and young adult women (Becker et al., 1999; Fairburn and Harrison, 2003; Walsh and Devlin, 1998). According to the American Psychiatric Association (2013), to be diagnosed with anorexia nervosa, the current diagnostic criteria that must be met for AN include: restricted food intake leading to weight loss or weight gain results in a significantly lower body weight than expected by age, sex, and height; fear of gaining weight or gaining weight; take a distorted view of yourself and your condition – for example, thinking that one is overweight despite being underweight, or believing one will gain weight by eating a single meal. The prevalence of AN is 2.2 to 4.0% (Keski-Rahkonen and Mustelin, 2016; Smink et al., 2013), and the incidence of AN in women aged 15 to 19 years is 270 per 100,000 (Keski-Rahkonen et al., 2007). Although there are a few long-term follow-up studies for AN, it tends to have a chronic course and has a high risk of mortality (Harris and Barraclough, 1998; Sullivan, 1995; Theander, 1985). Many international treatment guidelines of AN emphasize the importance of receiving professional treatment (Hilbert et al., 2017).

Two major problems with specialized treatment for anorexia nervosa exist. First, as with all eating disorders, many patients do not receive evidence-based treatment (Cooper and Kelland, 2015; Kazdin et al., 2017). According to a previous study of women in the United Kingdom, only 27.4% of patients diagnosed with an eating disorder have been got treatment in their lives (Micali et al., 2017), which may be due to few therapists adhering to evidence-based treatment protocols (Kosmerly et al., 2015; von Ranson et al., 2013; Waller, 2016). In a word, there are “research-clinical gap” in the treatment for eating disorders (Cooper and Bailey-Straebler, 2015; Fairburn and Wilson, 2013). With the development of ICT, the Internet usage rate rase and information and communication devices have been becoming widespread in developed countries, so self-help programs via Web site on the internet is a useful way to improve accessibility to treatment. Web program-based interventions are unaffected by the shortage of therapists and may solve the “research-clinical gap” problem described above.

Another problem is that short-term effective treatments for anorexia nervosa have not been established (Galsworthy-Francis and Allan, 2014), although one long-term prognosis study reported recovery in some patients after 5 years (Keski-Rahkonen et al., 2007). The treatment guidelines published by the National Institute for Health and Care Excellence (NICE) in the United Kingdom recommend cognitive-behavioral therapy (CBT), expert-supported clinical management, and Maudsley Model of Anorexia Nervosa Treatment for Adults (MANTRA) (National Institute for Health and Care Excellence, 2017). Although no specific therapeutic approach has been found to be the most effective in adults with AN (Byrne et al., 2017; Watson and Bulik, 2013), CBT may be a promising treatment (Calugi et al., 2017; Dalle Grave et al., 2016; Steinglass et al., 2014).

CBT aims to improve patient dysfunction by identifying cognitive and behavioral patterns that sustain clinical symptoms and encouraging the development of a variety of cognitive-behavioral skills. CBT has been shown to be not significantly different between face-to-face and therapeutic effects when performed with self-help programs (Cuijpers et al., 2019), include self-help via the Internet (Andersson et al., 2014; Carlbring et al., 2018). Internet-based cognitive-behavioral therapy (ICBT) has been shown to be effective for eating disorders other than anorexia nervosa (ter Huurne et al., 2015), suggesting that ICBT may prevent the recurrence of symptoms in those who have recovered from AN (Schlegl et al., 2015). In addition, ICBT for individuals caring for those with AN has begun to be implemented (Grover et al., 2011; Hoyle et al., 2013).

However, study of ICBT for patients with AN is limited. Therefore, in this study, we conducted a prospective single-arm clinical trial to investigate whether ICBT in patients with AN is safe and practical. In this study, we present the results of a pilot feasibility clinical study in Japan.

## 2 Methods

### 2.1 Study design and procedure

The present study was designed as a single-arm study of therapist-guided ICBT to investigate the feasibility of the intervention for patients with AN in Japan. The recruitment period was from January 6, 2020, to December 31, 2020, and the clinical trial period was from January 6, 2021, to March 31, 2021. Participants were recruited through several methods of advertisement, including a posting on the Chiba University website, an information portal site for eating disorders, Google advertisements, Facebook, and Instagram. These advertisements instructed applicants to access the participant recruitment form at Chiba University. Candidates who wished to participate in this study were contacted by the researchers, and the schedule was adjusted to allow participants to provide informed consent and be screened at Chiba University Hospital. After an initial screening at Chiba University Hospital, those meeting the eligibility criteria were directly handed a document containing user information and a password to access the ICBT program, which was the trial treatment.

The treatment period lasted 12 weeks (3 months), and the schedule was adjusted to allow the participants to be assessed after the intervention was completed at Chiba University Hospital. Those who qualified for the current trial completed a course of 12 ICBT modules, each of which lasted about 15 min. The primary and secondary endpoints for efficacy and safety assessments were performed at initial screening (baseline) and post-treatment (after approximately 3 months/12 weeks). The clinical trial protocol was reviewed and approved by the Ethics Review Committee of Chiba University Hospital (examination number: G2019018). Before the study commenced, the clinical trial protocol was registered at the Japanese clinical trial registration site (UMIN000039485).

### 2.2 Participants

Sixteen women met eligibility criteria: a) meeting AN in the Diagnostic & Statistical Manual of Mental Disorders, 5th ed. (DSM-5) diagnostic classification (American Psychiatric Association, 2013); b) being aged 15 to 65 years; c) receiving a standard treatment; d) having no plans to change medications or start new treatments during the study period; e) have access to telecommunications equipment to use the ICBT program; f) being able to send emails, chat, and have the necessary information and communication technology skills, and g) not receiving CBT in the last 2 years. Exclusion criteria involved those who were expected to interrupt CBT due to the following: organic brain disorders, psychotic disorders, drug addiction, antisocial personality disorders, acute stress disorder, post-traumatic stress disorder, hospitalized and at risk of physical complications, or having a serious safety risk (e.g., BMI < 12, imminent risk of suicide).

### 2.3 Intervention

**Fig. 1:**
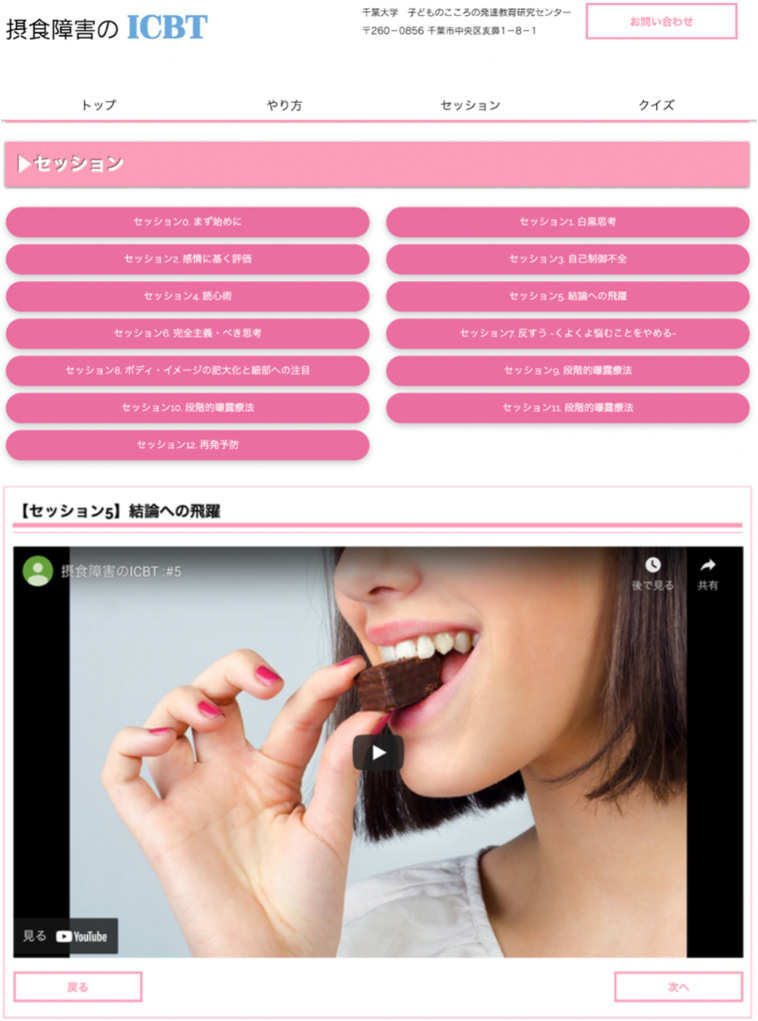
Website session page.

The first author (SH) and the second author (KM) implemented the ICBT program using multiple web platforms (Fig. 1). The intervention program was largely based on metacognitive training. Table 1 shows the weekly treatment modules and the total number of sessions. There were 12 treatment modules, and each module focused on one theme and was influenced by metacognitive training used to treat schizophrenia and depression (Jelinek et al., 2015; Moritz et al., 2014) since metacognitive vulnerabilities and schemas are often prominent in AN (Anderluh et al., 2003; Davenport et al., 2015; Hamatani et al., 2016; Smith et al., 2018). The cloud-based content management system service, “WIX,” established in Israel in 2006, was used to provide the ICBT. A medical chat service (medical SNS) developed by Share Medical Co., Ltd. that replaced a phone was used for interactions between the patient and the therapist. Patients completed the treatment content stored in WIX once a week at their convenience, and after completing the module, patients would contact the cognitive-behavioral therapy therapist (researcher) via Mediline®️ for feedback.

**Table 1:** Treatment modules of for the Internet-based cognitive behavioral therapy by week.

Week	Module
0	Guidance of the treatment for anorexia nervosa
1	Dichotomized thinking
2	Emotion-based evaluation
3	Self-control failure
4	Mind reading
5	Jumping to conclusion
6	Perfectionism
7	Rumination
8	Enlarged self-body image and attention to detail
9–11	Exposure and response prevention
12	Prevention of relapse

### 2.4 Quality control for ICBT

The therapist (SH) was a clinical psychologist/certified public psychologist who had a PhD in clinical psychology and specialized in eating disorders, including experience in providing face-to-face and video conferencing CBT to patients with eating disorders. The therapist had experience using the ICBT format when working with patients having panic disorder and obsessive-compulsive disorder. The quality of CBT was controlled by peer supervision between the first and second authors.

### 2.5 Measures

#### 2.5.1 Primary outcomes

The primary outcome was the severity of eating disorders as measured by the global score on the Eating Disorder Examination Questionnaire (EDE-Q; Fairburn and Beglin, 1994). The EDE-Q is a 28-item self-report questionnaire. Items are rated on a 7-point Likert scale (0–6), with a score of 4 or higher indicating clinical significance. The global EDE-Q score was calculated by dividing the sum of the four subscales (Restraint, Eating Concern, Shape Concern, and Weight Concern) by 4. The internal consistency of the EDE-Q was adequate, and the relationships with other measures demonstrated convergent validity (Mitsui et al., 2017).

### 2.6 Secondary outcomes

The EDE-Q subscales (Restraint, Eating Concern, Shape Concern, and Weight Concern) were used as secondary endpoints (Fairburn and Beglin, 1994). Further, the Body Shape Questionnaire (BSQ) was used to evaluate body image (Cooper et al., 1987; Mera et al., 2010). The BSQ is a 34-item self-report questionnaire rated on a 6-point scale (*never* to *always)* that evaluates the magnitude of attention to body shape and appearance and the feeling of being fat. The scale has previously demonstrated strong internal consistency (*α* = 0.97) and good validity (Kobayashi et al., 2001). The frequency of physical examination behaviors was measured using the Body Checking Questionnaire (BCQ), a 23-item self-report inventory (Reas et al., 2002). It is scored on a 5-point rating scale ranging from (*not*) to 5 (*very often*). The scale has demonstrated adequate validity and good reliability (Reas et al., 2002).

A 30-item short form of the metacognition questionnaire (MCQ-30) was used to measure metacognitive beliefs (Wells and Cartwright-Hatton, 2004). The MCQ-30 is a self-report instrument with items rating on a 4-point scale (*not applicable* to *very applicable*) and is designed to evaluate five metacognitive beliefs: Lack of Cognitive Confidence; Positive Beliefs about Worry; Cognitive Self-Consciousness; Negative Beliefs about Uncontrollability and Danger; Need to Control Thoughts. The scale's internal consistency, homogeneity, and validity have been established (Tazaki, 2017).

We measured the severity of depressed mood and generalized anxiety symptoms experienced in the last two weeks using the Patient Health Questionnaire (PHQ-9; Muramatsu et al., 2007; Spitzer et al., 1999) and the Generalized Anxiety Disorder Scale (GAD-7; Muramatsu, 2014; Spitzer et al., 2006), respectively. The PHQ-9 and GAD-7 are scored on a 4-point scale (0 = *none*, 1 = *a few days*, 2 = *more than half*, 3 = *almost daily*). The PHQ-9 scores range from 0 to 27, and the cutoff value for clinically significant depressive symptoms is 10. The PHQ-9 defines a symptomatology as a score ranging from 0 to 9, mild from 5 to 9, moderate from 10 to 14, moderate-to-severe from 15 to 19, and severe depressive state from 20 to 27. The Japanese version of the PHQ-9 has demonstrated adequate validity (Muramatsu et al., 2007). GAD-7 scores ranged from 0 to 21, and the cutoff value for clinically significant generalized anxiety was 10. The GAD-7 defined a symptomatology as a score from 0 to 4, mild from 5 to 9, moderate from 10 to 14, and severe general anxiety from 15 to 21. The GAD-7 had good reliability and validity (Spitzer et al., 2006).

Quality of life (QOL) was evaluated by calculating quality-adjusted life years using the EuroQol 5 dimensions 5-level questionnaire (EQ-5D-5L; EuroQol Group, 1990; Tsuchiya et al., 2002), a self-report scale with scores ranging from 0 (*death*) to 1 (*health*). The scale has shown both of validity and reliability (Feng et al., 2021). We also asked about the presence or absence of menstruation and measured body mass index (BMI) before and after the study treatment.

We used the Working Alliance Inventory-Short Form (WAI-SF), which assesses the strength of the therapeutic alliance (Tracey and Kokotovic, 1989), to assess the goodness of the therapeutic relationship between the therapist and the patient. The WAS-SF is a 12-item self-report questionnaire with items rated from 1 to 8 on a 7-point Likert scale. The higher the WAI-SF total, the better the therapeutic relationship and the stronger the treatment alliance with scores ranging from 12 to 84. The WAI-SF was administered only at the end of treatment. Convergent validity was shown and sufficient reliability was obtained (*α* = 0.93, 0.96) (Kawamura et al., 2020).

Patient satisfaction with the treatment and guided ICBT was also assessed using the Client Satisfaction Questionnaire (CSQ; Attkisson and Zwick, 1982; Tachimori and Ito, 1999). Finally, blood data were collected in advance at baseline and after treatment. Sufficient internal consistency (*α* = 0.83) and constant criteria-related validity (*r* = 0.36 to 0.49) were established (Tachimori and Ito, 1999).

### 2.7 Adverse events and safety

We defined any unfavorable or unintended signs (including abnormal laboratory test values), symptoms, or illnesses during the study protocol as adverse events in the current clinical trial. We confirmed the presence or absence of adverse events at Chiba University Hospital using MediLine®︎ from patients after each ICBT module was completed to evaluate patient safety and the occurrence of adverse events.

### 2.8 Statistical analysis

All participants enrolled in the study who completed one or more sessions of ICBT and had efficacy data were included in the analysis as the largest population to be analyzed (FAS). Statistical analyses were performed using the SAS statistical software package, Version 9.4 (SAS Institute, Cary, NC, USA). Summary statistics (mean, median, standard deviation, minimum, maximum) and effect size before and after the intervention (Cohen's *d*) were calculated. A paired *t*-test was used to compare scores before and after the intervention. An alpha level of 5% was set as the significance level, and 95% confidence intervals were calculated. In this study, the effect size was calculated from the obtained change amount using the following formula to determine the number of samples when conducting future ICBT randomized controlled trials (Cohen's *d*, where ∆ = effect size; *μ* = mean of value difference; *Sd* = difference in standard deviation). A Cohen's *d* > 0.20 was used as the criterion for a small effect, a value >0.50 as a medium effect, and >0.80 as a large effect (Cohen, 1988).

For the primary analysis, we considered the difference between the EDE-Q scores at the baseline and endpoint. We analyzed the change in the primary and secondary outcomes to supplement the main primary analyses and examine effectiveness. For categorical variables, the McNemar test was used for two categories. The frequency for the adverse events were calculated as a safety analysis.

Regarding the sample size design, we adopted the recommended number of cases of 12 when little information was available from previous studies (Julious, 2005). In the current clinical trial, the target number of patients who completed the study treatment was 12, and the total number of registered cases was set to 17, given a predicted dropout rate of 30%.

### 2.9 Role of funding source

This study was supported by JSPS KAKENHI (grant numbers: 18K17313; 19J00227). The funding sources had no role in the study design, collection, analysis, and interpretation of data; in the writing of the report; and in the decision to submit the article for publication.

## 3 Results

### 3.1 Recruitment

**Fig. 2:**
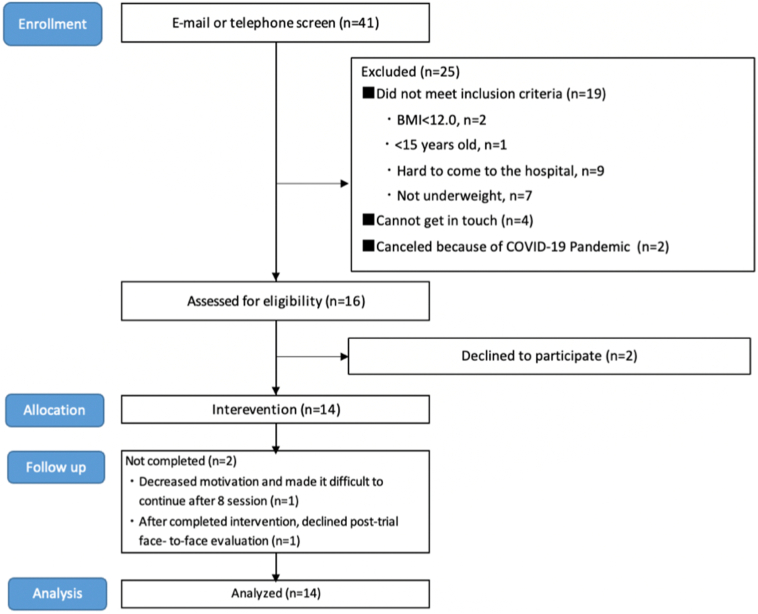
Participant flow chart for feasibility study that shows the number of cases included from recruitment to analysis and the reasons for withdrawal from this study. Forty-one individuals were recruited. Ultimately,14 people started trial treatment and 2 dropped out. Analyses included the 14 cases who began the treatment program.

Fig. 2 shows a flow diagram of participant recruitment. Of the 41 applicants, 16 met the eligibility criteria after completing email or telephone screening and were invited to study treatment. Following registration, two patients declined in the current clinical trial before the start of the intervention. Fourteen implemented the guided ICBT program at least one session and 13 completed all treatment modules. One of the 14 patients dropped out of the intervention at the end of the eight sessions: we could measure EDE-Q and BMI by telephone assessment at the time. Twelve of the other patients completed assessment session at post-intervention, one patient declined the assessment. Therefore, data from 14 patients at baseline and 12 patients (13 patients for only EDE-Q and BMI) after the study completion were included in the analysis.

### 3.2 Participant characteristics

The demographic data of this study are presented in Table 2. The 14 women who participated had a mean age of 29.7 years (*SD* = 10.8, range: 15–51). Their average BMI at baseline was 14.9 (*SD* = 1.6, range: 12.0–17.3). The mean of estimated IQ on the JART (Matsuoka et al., 2002; Nelson and O'Connell, 1978) was 109.9 (*SD* = 7.4, range: 94–118). The mean years of education was 14.0 (*SD* = 2.6, range: 9–18). Five out of 14 patients were employed full-time, two part-time, and their mean years of employment was 6.4 (*SD* = 8.7, range: 0–30). The patients treated in this study seem to represent typical AN—young women with above-average developmental intelligence and clinically meaningful underweight. Seven (50.0%) were currently taking psychotropic medication: Sulpiride (*n* = 2), Ramelteon (*n* = 2), Etizolam (*n* = 1), Brotizolam (*n* = 1), Levomepromazine Maleate (*n* = 1), and Aripiprazole (*n* = 1).

**Table 2:** Demographic and clinical characteristics (*N* = 14).

Variable	*M* (*SD*)
Age (years)	29.7 (10.8)
Education (years)	14.0 (2.6)
BMI (kg/m^2^)	14.9 (1.6)
Estimated IQ on the JART	109.9 (7.4)
	*n* (%)
Employment, has a job	7 (50.0)
Marital status, has a partner	1 (7.1)
Comorbidity	
Major depressive disorder	4 (28.6)
Generalized anxiety disorder	2 (14.3)
Past history	
Social anxiety	1 (7.1)
Family history of depression (yes)	4 (28.6)
Anorexia nervosa sub-type	
Restricted	6 (42.9)
Binge eating/purging	8 (57.1)
Combined pharmacotherapy	7 (50.0)

*Note*. *M*, mean; *SD*, standard deviation; JART, Japanese Adult Reading Test.

### 3.3 Primary and secondary outcomes

Global EDE-Q scores significantly decreased from baseline (*M* = 3.48, *SD* = 1.4) to post-intervention (*M* = 2.54, *SD* = 1.5; *p* = 0.02, Cohen's *d* = 0.75). Table 3 shows the mean changes in all the outcomes. For the subscales of the EDE-Q, eating concern (*p* = 0.01), shape concern (*p* = 0.02), and weight concern (*p* = 0.01) showed significant improvement. Body checking rated on the BCQ showed a significant reduction (*p* = 0.04), as did GAD-7 scores (*p* = 0.01). At the time of the final session (i.e., Week 12), the average total WAI-SF was 67.9 (*SD* = 10.3), and the average total satisfaction was 25.4 (*SD* = 3.6). Guided ICBT-style interventions appeared to have established a strong therapeutic alliance between the therapist and the patient and were accepted by the patient. In addition, after treatment, one patient did not meet the diagnostic criteria for AN with DSM-5 (American Psychiatric Association, 2013).

**Table 3:** Changes in outcomes from pre- to post-test (*N* = 14).

Outcomes	Time point	*n*	*M* (*SD*)	Cohen's *d*	95% *CI*	*p-*Value
Eating Disorder Examination Questionnaire						
Global score				0.75	[−1.75, −0.18]	0.02
	Pre	14	3.48 (1.42)	–	–	–
	Post	13	2.54 (1.51)	–	–	–
Restraint				0.44	[−1.94, 0.31]	0.14
	Pre	14	3.56 (2.02)	–	–	–
	Post	13	2.88 (1.85)	–	–	–
Eating concern				0.92	[−1.89, −0.39]	0.01
	Pre	14	3.00 (1.58)	–	–	–
	Post	13	1.87 (1.41)	–	–	–
Shape concern				0.75	[−1.86, −0.19]	0.02
	Pre	14	3.57 (1.49)	–	–	–
	Post	13	2.49 (1.45)	–	–	–
Weight concern				0.84	[−2.24, −0.37]	0.01
	Pre	14	3.30(1.76)	–	–	–
	Post	13	1.96 (1.49)	–	–	–

MCQ-30						
Total score				0.26	[−9.59, 4.09]	0.40
	Pre	14	76.21 (17.11)	–	–	–
	Post	12	73.83 (18.16)	–	–	–
1. Lack of cognitive confidence				0.06	[−2.04, 1.71]	0.85
	Pre	14	11.36 (4.70)	–	–	–
	Post	12	11.67 (4.01)	–	–	–
2. Positive beliefs about worry				0.13	[−1.64, 2.47]	0.66
	Pre	14	16.86 (4.13)	–	–	–
	Post	12	17.67 (4.91)	–	–	–
3. Cognitive self-consciousness				0.44	[−4.49, 0.82]	0.16
	Pre	14	17.29(3.81)	–	–	–
	Post	12	15.50 (3.99)	–	–	–
4. Negative beliefs about uncontrollability and danger				0.17	[−1.98, 1.15]	0.57
	Pre	14	17.00 (5.64)	–	–	–
	Post	12	16.00 (4.82)	–	–	–
5. Need to control thoughts				0.21	[−3.03, 1.53]	0.49
	Pre	14	13.71 (4.92)	–	–	–
	Post	12	13.00 (5.10)	–	–	–
Body Mass Index				0.44	[−0.09, 0.57]	0.14
	Pre	14	14.89 (1.64)	–	–	–
	Post	13	14.94 (1.63)	–	–	–
Body Checking Questionnaire				0.68	[−23.98, −0.85]	0.04
	Pre	14	65.43 (28.31)	–	–	–
	Post	12	55.42 (26.67)	–	–	–
Body Shape Questionnaire				0.59	[−43.51, 1.51]	0.06
	Pre	14	110.57 (38.17)	–	–	–
	Post	12	89.75 (38.61)	–	–	–
PHQ-9				0.59	[−6.40, 0.23]	0.07
	Pre	14	13.14 (6.16)	–	–	–
	Post	12	9.58 (4.87)	–	–	–
GAD-7				0.95	[−6.68, −1.32]	0.01
	Pre	14	10.57 (5.27)	–	–	–
	Post	12	6.00 (3.72)	–	–	–
EQ-5D-5L				0.55	[−0.01, 0.14]	0.08
	Pre	14	0.74 (0.11)	–	–	–
	Post	12	0.81 (0.11)	–	–	–

*Note*. *M*, mean; *SD*, standard deviation; *CI*, confidence interval; Body Mass Index is calculated by [weight (kg)]/[height (m)^2^]; MCQ-30, the 30-item short-form of the Meta-Cognition Questionnaire; PHQ-9, Patient Health Questionnaire; GAD-7, Generalized Anxiety Disorder Scale; EQ-5D-5L, EuroQol 5 dimensions 5-level.

### 3.4 Adverse events

No adverse events were identified during the study period.

## 4 Discussion

### 4.1 Principal findings

The prospective single-arm clinical trial aimed to investigate the safety and feasibility of guided ICBT in treating patients with AN. Fourteen women diagnosed with AN began the trial, and 12 of them completed the trial. The severity of AN symptoms on the EDE-Q global scores significantly improved from baseline to after the trial. The therapist's guide was provided through a chat tool, but the therapeutic relationships were well-established and confirmed to be highly satisfying using this form of intervention. No adverse events were observed. The results of the current trial suggest that guided ICBT is a potentially promising treatment for women with AN.

### 4.2 Comparison to previous studies

Some measures of AN severity showed significant improvement in the present study, suggesting the potential benefits of this guided ICBT. The significant improvement on the global scores on the EDE-Q, and the effect size was medium (Cohen's *d* = 0.75). A previous study reported that Exposure and Response Prevention (ERP) therapy improved dietary fear and increased caloric intake in patients with AN (Steinglass et al., 2014). In the present study, anxiety symptoms measured by the GAD-7 were significantly improved from baseline to after the study treatment, and the severity of eating disorders assessed by the global score of EDE-Q decreased, consistent with previous results (Calugi et al., 2017; Dalle Grave et al., 2016; Steinglass et al., 2014). CBT has shown limited efficacy for patients with AN compared to other eating disorders as bulimia nervosa or binge eating disorder (Galsworthy-Francis and Allan, 2014; Linardon et al., 2018), and weakness of cognitive functioning in patients with AN may affect treatment outcomes (Harper et al., 2017). In the present study, the treatment module focusing on AN metacognitive dysfunction was placed in the first half of the program. After this module, the program was structured to address ERP. The evidence from our clinical trial may indicate that the improvement in the metacognitive function contributed to the improvement of clinical symptoms and reduction of the dropout rate. However, no significant differences in the metacognitive functions on the MCQ-30 were observed, although the score decreased.

Patients with AN have comorbid conditions, such as depression and anxiety (Marucci et al., 2018; Woodside and Staab, 2006). There were marginal improvements in depressive symptoms on the PHQ-9 (Cohen's *d* = 0.59, *p* = 0.07), while self-reported symptoms of generalized anxiety significantly improved (Cohen's *d* = 0.95, *p* = 0.01). The present study is the first to report that a guided ICBT program for patients with AN may potentially improve symptoms of depression and anxiety.

Our results of the present study on dropout rate and completion rate include important findings into guided ICBT in patients with AN. Twelve of the 14 patients completed the current clinical trial and 2 dropped out. One patient of the 2 patients dropped out during the intervention program after reporting feeling “dis-motivated.” This patient had high depressive symptoms at baseline. In ICBT format, the severity of depressive symptoms increases the risk of dropout (Fernandez et al., 2015). Previous research has suggested that most patients with eating disorders (94%) can complete Internet-based interventions, even therapist-guided ICBT (ter Huurne et al., 2015). Our results of the current clinical trial are consistent with finding of those previous studies. That is, we found that depressive symptoms in patients with AN predicted dropout from guided ICBT, and that most patients with AN were able to complete guided ICBT.

Our findings suggest that the guided self-help program can establish high patient satisfaction and a strong therapeutic alliance (Knaevelsrud and Maercker, 2007). A Japanese study reported that patients with psychiatric disorders had been found to prefer remote interventions via videoconference to face-to-face conditions (Matsumoto et al., 2018); however, there are few reports of therapeutic relationships with guided ICBT in patients with AN. Thus, this finding is novel.

### 4.3 Strengths and limitations

The current clinical trial results present some novel information regarding treatment within the field of AN psychotherapy. The present study demonstrated the feasibility of guided ICBT with patients with AN who were psychiatric outpatients and is the first study of its kind globally. The CBT module that focuses on metacognitive function indicated a reduction of the clinical symptoms of AN.

There are some limitations to the present study. First, the current clinical trial conducted in this study was single-armed and did include a control group; thus, the effectiveness of guided ICBT for AN could not be investigated. A randomized control trial should be conducted to verify the efficacy of guided ICBT in treating AN. Second, patients' pharmacotherapy was not controlled for in the study analyses. Research indicates CBT has an additional effect when drug therapy is added (Hofmann et al., 2012; Reas and Grilo, 2008). Therefore, future research that includes pharmacotherapy should be conducted. Third, this study examined only the short-term symptomatologic improvement over three months and did not examine long-term prognosis. We plan to follow up with the patients in this study in the next year and investigate their long-term outcomes. Fourth, the study provided a trial treatment that was additional treatment for patients currently receiving standard treatment. Therefore, the practicality and effectiveness of guided ICBT alone are beyond the scope of this study.

### 4.4 Conclusions

Our finding demonstrated that guided ICBT to treat AN is well accepted by female patients and practical as a telemedicine approach aimed at improving symptoms. The therapist's guide may allow most female patients to complete the ICBT's self-help program while minimizing the rate of withdrawal from trial treatment. CBT that targets metacognition may improve metacognitive function and reduce the severity of AN and associated comorbidities, such as depression and anxiety.

## Trial registration number

UMIN000039485 (URL: https://upload.umin.ac.jp/cgi-open-bin/ctr/ctr_view.cgi?recptno=R000045023).

## Data availability statement

If you would like to use the data obtained from the study, please contact the corresponding author.

## Declaration of competing interest

None.

## Appendix A Supplementary data

Supplemental File Blood data. Supplemental File
