# Supplementary material for: Feasibility of guided internet-based cognitive behavioral therapy for patients with anorexia nervosa
Source: Internet Interv. 2022 Feb 14;27:100504. doi: 10.1016/j.invent.2022.100504 (PMC8897312; doi:10.1016/j.invent.2022.100504)
Supplement: Supplemental File — Blood data. [file mmc1.docx]

Supplemental File

Blood data

| **Outcomes** | **Time Point** | ***N*** | **Mean** | **SD** | **95%CI** | | ***p*** |
| --- | --- | --- | --- | --- | --- | --- | --- |
| ALB |  |  |  |  | -0.39 | 0.19 | .46 |
|  | Pre | 14 | 4.59 | 0.30 | ― | ― | ― |
|  | Post | 12 | 4.47 | 0.36 | ― | ― | ― |
| ALT |  |  |  |  | -21.23 | 19.83 | .23 |
|  | Pre | 14 | 25.36 | 16.47 | ― | ― | ― |
|  | Post | 12 | 25.50 | 15.11 | ― | ― | ― |
| ALP* |  |  |  |  | -27.37 | 26.87 | .98 |
|  | Pre | 10 | 182.10 | 54.05 | ― | ― | ― |
|  | Post | 8 | 198.63 | 58.90 | ― | ― | ― |
| ALP** |  |  |  |  | -59.68 | 54.68 | .68 |
|  | Pre | 2 | 54.00 | 14.14 | ― | ― | ― |
|  | Post | 2 | 51.50 | 7.78 | ― | ― | ― |
| AST |  |  |  |  | -1.60 | 7.43 | .18 |
|  | Pre | 14 | 28.64 | 7.56 | ― | ― | ― |
|  | Post | 12 | 30.92 | 12.68 | ― | ― | ― |
| Amy |  |  |  |  | -35.86 | 11.03 | .27 |
|  | Pre | 14 | 147.50 | 68.88 | ― | ― | ― |
|  | Post | 12 | 142.50 | 49.45 | ― | ― | ― |
| γ‐GTP |  |  |  |  | -5.96 | 3.46 | .57 |
|  | Pre | 14 | 26.14 | 18.44 | ― | ― | ― |
|  | Post | 12 | 20.33 | 15.82 | ― | ― | ― |
| Hb |  |  |  |  | -0.51 | 0.51 | > .99 |
|  | Pre | 13 | 12.65 | 0.74 | ― | ― | ― |
|  | Post | 11 | 12.69 | 0.59 | ― | ― | ― |
| K |  |  |  |  | -0.11 | 0.38 | .26 |
|  | Pre | 13 | 3.93 | 0.36 | ― | ― | ― |
|  | Post | 12 | 4.10 | 0.36 | ― | ― | ― |
| Na |  |  |  |  | -0.69 | 1.19 | .57 |
|  | Pre | 13 | 139.54 | 3.97 | ― | ― | ― |
|  | Post | 12 | 139.92 | 3.80 | ― | ― | ― |
| PLT |  |  |  |  | -28.48 | 2.48 | .09 |
|  | Pre | 13 | 246.54 | 56.36 | ― | ― | ― |
|  | Post | 12 | 252.25 | 54.20 | ― | ― | ― |
| TP |  |  |  |  | -0.39 | 0.26 | .66 |
|  | Pre | 14 | 7.18 | 0.38 | ― | ― | ― |
|  | Post | 12 | 7.09 | 0.41 | ― | ― | ― |
| WBC |  |  |  |  | -0.83 | 0.41 | .47 |
|  | Pre | 13 | 4.40 | 0.98 | ― | ― | ― |
|  | Post | 12 | 4.38 | 1.32 | ― | ― | ― |

*Note.* ALB is albumin, a value that represents nutritional status; ALT is alkaline phosphatase which values that represent liver damage; ALT is alanine aminotransferase, which values that represent liver state; AST is aspartate aminotransferase, which values that represent liver state and the condition of the heart; Amy is amylase, typical value of inflammation of pancreas and salivary glands; γ-GTP isγ‐glutamyl transpeptidase, which values that represent liver condition; Hb is hemoglobin as an indicator of symptoms such as anemia or hypercytosis; K is potassium as an indicators of dehydration associated with laxative use and vomiting; Na is sodium, as an indicators of dehydration associated with laxative use and vomiting; PLT is platelet, used for diagnosing bleeding tendency, blood coagulation diseases; TP is total protein and represents liver function and nutritional status; WBC is white blood cell, the value fluctuates when there is inflammation in the body or when there is a blood disorder. * ALP_JSCC, **ALP_IFCC
